# A coarse-grained model of the expansion of the human rhinovirus 2 capsid reveals insights in genome release

**DOI:** 10.1098/rsif.2019.0044

**Published:** 2019-08-14

**Authors:** Giuliana Indelicato, Paolo Cermelli, Reidun Twarock

**Affiliations:** 1 Department of Mathematics, University of York, York, UK; 2 Department of Mathematics, University of Turin, Turin, Italy

**Keywords:** minimum energy paths, viral structural transitions, energy landscape

## Abstract

Human rhinoviruses are causative agents of the common cold. In order to release their RNA genome into the host during a viral infection, these small viruses must undergo conformational changes in their capsids, whose detailed mechanism is strictly related to the process of RNA extrusion, which has been only partially elucidated. We study here a mathematical model for the structural transition between the native particle of human rhinovirus type 2 and its expanded form, viewing the process as an energy cascade, i.e. a sequence of metastable states with decreasing energy connected by minimum energy paths. We explore several transition pathways and discuss their implications for the RNA exit process.

## 1. Introduction

Human rhinovirus (HRV) is the cause of the common cold that affects billions of people each year. The small viral particle, about 30 nm in diameter, has a protein shell, called the viral capsid, that encapsulates and thus protects its single-stranded RNA genome. An essential step in the infection process is the structural rearrangement of the proteins in the capsid shell, as this rearrangement results in the formation of pores through which the genomic RNA is extruded during the infection. A better understanding of this mechanism may therefore point to novel targets for anti-viral therapy and prevention.

**Figure 1.**
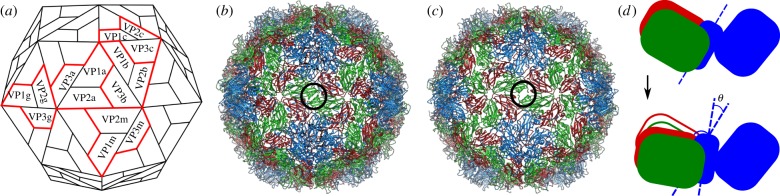
The capsid. (*a*) Sketch of the arrangement of the coat proteins VP1–VP3 in the native virion. Small-case Latin letters label coat proteins in the same protomer: 5 protomers are highlighted, labelled by (*a*, *b*, *c*, *g*, *m*). (*b*) The HRV2 native particle (PDB ID 1fpn). VP1s are displayed in blue, VP2s in green and VP3s in red. The black circle at the centre of the figure highlights a twofold position where pores are going to open as a consequence of protein displacement. (*c*) The expanded particle (PDB ID 3tn9—same colour coding as in (*b*) applies). (*d*) Details of the hinge movement within the protomer due to the relative rotation of the subunits of VP1 (adapted from [10]): VP1, VP2 and VP3 are coloured in blue, green and red, respectively. (Online version in colour.)

Conformational changes occur in a number of viruses during infection [1–4]. In each case, the spatial rearrangement of coat protein or coat protein domains results in the expansion of the capsid and the opening of pores through which the genomic material, and in some cases also other viral components, are released. We focus here on the pseudo *T* = 3 icosahedral capsid of HRV type 2 (HRV2), which is made of 60 protomers, each composed of four coat proteins VP1 to VP4 (figure 1*a*). While the three larger proteins (VP1, VP2, VP3) form the exterior surface of the particle, the smaller VP4 is located at the interior capsid surface. A characteristic feature of the protomer is the presence of a hydrophobic pocket located at the core of VP1, that in the native virus is occupied by the so-called pocket factor, presumably a fatty acid, which is believed to stabilize the capsid [5–10].

During infection, HRV attaches to the membrane of the cell at a receptor site, is internalized into the endosome, where it loses its pocket factors and the capsid expands, leading to the formation of the pores. The RNA exits the capsid through pores at the twofold axes and enters the host cell through channels in the endosomal membrane [11]. The mechanism by which RNA is released has not been completely elucidated, but experimental evidence suggests that exit occurs by an organized mechanism and is preceded by a substantial reorganization of the RNA inside the capsid [12]. The details of the conformational changes leading to the expansion of the capsid and the opening of the pores are likely to be related to the mechanism by which RNA is released. Moreover, it is known that for some viruses belonging to the same genus of HRVs, i.e. the Enterovirus genus, the engagement with the receptor triggers the conformational change [13–16].

In addition to the phylogenetic classification HRVs are divided into a major group and a minor group based on which cellular receptor they use for viral entry: major-group HRVs bind to the intercellular adhesion molecule 1 receptor, while the minor-group HRVs bind the low-density lipoprotein receptor. In the major group, the conformational changes of the capsid leading to RNA release are triggered by the interaction with the cellular receptor, whereas in the minor group, the low endosomal pH induces the removal of the pocket factor and the consequent viral structural rearrangements that leads to the expansion of the capsid.

We focus here on HRV2, a minor group rhinovirus, and we study the transition pathways between the native particle (figure 1*b*) and its genome-containing expanded form, the A-particle, which is a metastable intermediate on the pathway to the empty B (80S) particle (figure 1*c*). During this transition, individual protomers of the capsid undergo a conformational change, during which the two domains of VP1 move apart by an angle *θ* (figure 1*d*; called opening event in the following), and VP2 and VP3 move in concert with one of the domains. Opening of all protomers collectively results in expansion of the native particle by about 4% in radius and release of the VP4s.

The mathematical model used here is based on the coarse-grained approach developed in [17], in which the capsid proteins (CPs) are viewed as elementary units interacting by weak bonds, and the resulting energy landscape is explored by determining the paths and the energy barriers joining the metastable states. This allows to study the order in which the individual protomer transitions might occur, in order to identify the likely transition kinetics of the capsid expansion event.

In particular, we have addressed two issues that are relevant for understanding genome release, an essential part of the infection process (for more details, see the Discussion section).

First, we have investigated whether the structural transition of the capsid during its expansion is governed by diffuse nucleation events, with no regularity, or by a more organized domino effect, as suggested by the fact that interactions between the capsomers are relatively weak (the energy cascade hypothesis).

Second, and more importantly, given that it has been experimentally established that (i) RNA exit is directional, with the 3^′^-end exiting first, and (ii) the positions of the 5^′^- and 3^′^-ends inside the capsid are fixed in areas of the capsid that are roughly opposite to each other [18,19], the following question arises: What is the relation between the localization of the 3^′^-end and the opening of the pores in the capsid and in the endosome through which the 3^′^-end exits? Our model has been designed to address this issue. In particular, we explore whether there are pathways that induce a preferential opening of the pores near the 3^′^ site, and we show that there is a reasonable parameter range in which this is the case. In fact, there is a clear separation in the parameter space between three possible modes of opening, and this suggests that a fine regulation of the RNA-extrusion process (i.e. the preferential opening of a twofold channel in the capsid near the attachment site of the 3^′^-end of the RNA) is possible without requiring regulation via the action of receptors. This is consistent with the fact that some strains of human rhinovirus, such as HRV2 that is under consideration here, do not require receptors for genome release [11], and suggests a principle of economy in the release mechanism.

Note that, in order to be effective, the above mechanism requires that the cascade is triggered at the protomer at which the 3^′^-end of the RNA is bound. This could perhaps be triggered by a specific interaction between genomic RNA and CP at that site, that impacts on that CP’s conformation and its interactions with surrounding CPs. Conformational changes in CP in response to contact with an RNA stem-loop have been reported in other viruses before, such as the allosteric conformer switch in MS2 that is a prerequisite for capsid assembly [20,21]. Also, in human parechovirus (HPeV), a different picornavirus that does not cleave its VP0 into VP2 and VP4, we have recently shown that there are multiple dispersed RNA sequence/structure motifs in the viral genome with affinity for CP that we termed packaging signals due to their role in capsid formation [22,23]. Cryo-electron microscopy studies of rhinovirus also show multiple dispersed contacts between genomic RNA and capsid, with a change in the contact pattern upon expansion [12]. In particular, in addition to the contacts close to the twofold axes that are present already in the native HRV2 particle, there are new contacts around the fivefold axes in the expanded A-particle (cf. fig. 2 in [12]). It is, therefore, possible that contacts between genomic RNA and CP play a role in the release mechanism.

## 2. A model of the structural transition

During infection, HRV2 is internalized by the cell within endosomes, and the decrease in pH triggers a series of structural transformations of the capsid, leading to the expansion of the particle, the formation of pores and to the exit of the viral RNA into the cytosol. The particle has been imaged at different stages of the expansion: the structure of the native particle has been determined at 2.6 Å resolution by X-ray crystallography [6], while X-ray crystal structures at 6.4 Å and cryo-electron microscopy studies [12] showed that A (the genome-containing expanded form—see the Introduction) and B (the expanded and empty form) particles are almost identical, and an X-ray structure has been determined at 3.0 Å resolution for the empty particle [10]. Henceforth, in what follows, we identify the A capsid with the B capsid.

The full dynamics of the process is still unclear, although some of the occurring structural transformations have been in part elucidated. It is generally acknowledged that, as a consequence of the decrease in pH, the pocket factor at the core of VP1 is released from its location, and this allows the relative rotation of two domains of VP1 (the *α* helix and the C-terminus move away from the *β*-barrel), resulting in a change in the conformation of the monomer and the collapse of the pocket. This hinge movement affects VP2 and VP3 positions as they displace in concert with one of VP1 domains [7,8,11].

The relative motion of the subunits of VP1 together with VP2, VP3 leads to an increase of the diameter of the capsid, and results in the opening of three types of pores, two of which at the quasi-threefold and twofold axes. The other channels are located at symmetry-related positions (around the fivefold axes) and supposedly allow to externalize a portion of VP1 (N-terminal residues) that is thought to be instrumental in the adhesion of the capsid to the interior of the endosomal membrane, through which the RNA must pass in order to enter the cytosol.

The pores at the twofold axes are relevant for infection because they are wide enough to allow the transit and exit of VP4 and the RNA. With reference to figure 1*a* they are located at the interfaces VP2a:VP2m and at positions related by icosahedral symmetry, and their formation is caused by the relative clockwise motion of VP2a and VP2m that move away from each other as a result of the hinge movement within the protomers [10]. There is evidence that the RNA leaves the capsid at one of the pores at the twofold axes [24,25], and the process starts at its 3^′^ end [18]. Furthermore, in the native virion, the 5^′^ end, which is the end at which RNA synthesis begins, is bound inside the capsid to a CP at a threefold site roughly opposite the twofold exit side.

### 2.1. The coarse-grained model

**Figure 2.**
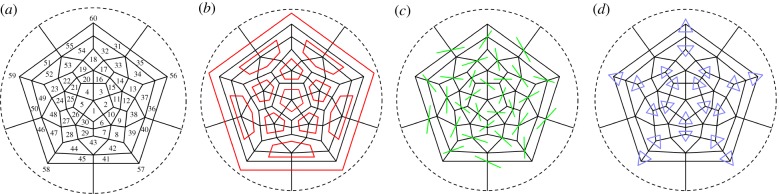
(*a*) Schlegel diagram of the capsid architecture, showing the numbering scheme used to identify individual protomers in the viral capsid. (*b*) Intra-pentamer interactions around the particle fivefold axes (adjacency matrix *A_ij_*) are shown as pentagons (red in the online version). (*c*) Inter-pentamer interactions across the particle twofold axes (adjacency matrix *B_ij_*) are shown as lines (green in the online version). (*d*) Inter-pentamer interactions around the particle threefold axes (adjacency matrix *C_ij_*) are shown as triangles (blue in the online version). (Online version in colour.)

The expansion of the capsid is the result of a collective rearrangement induced by rigid body motions of protomer domains (figure 1*b*,*c*,*d*). These rigid motions will be parametrized by a single angular variable, one for each protomer, denoted by *θ*_*i*_ ∈ [0, 1] for *i* = 1, …, 60, with the convention that *θ*_*i*_ = 0 and *θ*_*i*_ = 1 correspond to the closed and open configuration of the protomer, respectively. Also, we assume that the protomers are labelled as in figure 2*a* on a Schlegel diagram of the capsid. The numbering scheme has been chosen such that protomers in the same pentamer are consecutively numbered, and that the numbers of opposite protomers add up to 61. Each capsid configuration is associated with a vector ***θ*** = (*θ*_1_, …, *θ*_60_). Hence, the two states $\theta_{i}=0\;\forall i$ and $\theta_{i}=1\;\forall i$ correspond to the native (closed) and expanded (open; A particle) capsid.

The changes in the capsid may occur either simultaneously or as a cascade of subsequent events, and we model it as a sequence of elementary transitions between metastable states, i.e. local minima, of a suitable energy function. The energy cascade mechanism has been demonstrated for the maturation of HK97 in [26,27], and mathematical models based on these ideas have been developed in [17,28].

We assume that the free energy is the sum of three components, that take protein–protein interactions into account, and model protein–RNA interactions as parameters modifying these energy terms. The first term, that we call the intra-protomer energy, accounts for the forces that drive the structural transition of a protomer after the removal of the pocket factor, as well as the barrier that has to be overcome to break the bonds necessary for this process. The second term is the intra-pentamer energy, that captures contributions from the bonds between the adjacent protomers within the same pentamer. Finally, the inter-pentamer energy accounts for the bonds between adjacent protomers belonging to different pentamers.

The above terms only account for the energy barriers related to the breaking of bonds between different capsomers (or domains of the same protomer, as is the case for VP1). However, the interactions between the capsomers are much more sophisticated, and certainly include mechanical knock-on effects due to their mutual push and pull during the opening of the protomers and the expansion. To include these effects, we introduce a weak constraint on the motion of adjacent capsomers via a penalization term in the total energy.

### 2.2. Intra-protomer interactions

The relative rotation of the subunits of VP1 upon removal of the pocket factor has the effect of widening the angle between the domains by *θ*, that we will call the opening angle in the following, thereby disrupting and forming a number of bonds between the subunits. The impact of this conformational change on the protein interfacial energies has been quantified in [10] (see also table 1).

**Table 1.** Changes in protein–protein interactions during the transition from native to 80S particle. The table and its description are adapted from the electronic supplementary material in Garriga *et al.* [10], and the labelling of the proteins is as in figure 1*a*. The number of unique residue pairs that contact each other at the subunit interface is listed for each interface in the native and the 80S particles. The numbers of mutual interactions in both capsids are listed in the fifth column. The sixth and seventh column indicate the number of interactions that are only present in the native and in the 80S particle, respectively. The association energies (kcal mol^−1^) for each interface of each capsid type are stated in the eighth and ninth columns. The interacting residue pairs, association energies for each interface were calculated using VIPERdb tools.

		interactions					association energy	
		native	80S	conserved	lost	new	native	80S
intra-protomer	VP1a–VP2a	82	75	71	11	4	−84.7	−70.2
	VP1a–VP3a	151	110	96	55	14	−188.8	−142
	VP2a–VP3a	66	62	57	9	5	−68.9	−68.5
intra-pentamer	VP1a–VP1b	44	26	19	25	7	−40.7	−33.4
	VP1a–VP3b	42	28	20	22	8	−56.9	−38.8
	VP2a–VP1b	14	0	0	14	0	−16.7	0
	VP2a–VP3b	33	20	16	17	4	−28.1	−27.5
	VP3a–VP1b	10	7	7	3	0	−12.5	−3.9
	VP3a–VP3b	33	30	29	4	1	−41.1	−38
	VP3a–VP3c	4	4	4	0	0	−3.6	−3.1
inter-pentamer	VP1a–VP2g	20	0	0	20	0	−20.3	0
	VP3a–VP2g	63	49	33	30	16	−54.6	−47.4
	VP2a–VP2m	14	6	1	13	5	−26.5	−11.4

We describe the switch between the two configurations of the protomer via a two-well energy function, with minima at the closed and open configuration of the protomer. It is this energetic term that drives the expansion of the capsid in our model.

To motivate our choice of two-well energy, we note that while in other viruses (cowpea chlorotic mottle virus, equine rhinitis A virus (ERAV)) there is evidence that the confinement of the RNA inside the capsid induces an internal pressure that drives the expansion, no evidence for such a mechanism is present for HRV2. Rather, the idea here is that, after the removal of the pocket factor, the domains of VP1 within a protomer are free to attain a preferred stable configuration, which suggests that the intra-protomer energy must have a minimum *h* < 0 in correspondence of this metastable state (*θ* = 1).

However, as mentioned above, in order to reach this state the two domains of VP1 have to break a number of bonds, which suggests that the closed form of the protomer is metastable (hence the first minimum at *θ* = 0) and that there is a barrier *k* between these two wells whose size is proportional to the energy necessary to break those bonds.

Finally, we know that (for values of the pH as in the endosome and in the absence of the pocket factor) the open capsid is more stable than the closed one, which suggests that the minimum at *θ* = 1 is deeper than the minimum at *θ* = 0, so that the reverse transition is more difficult than the forward one.

**Figure 3.**
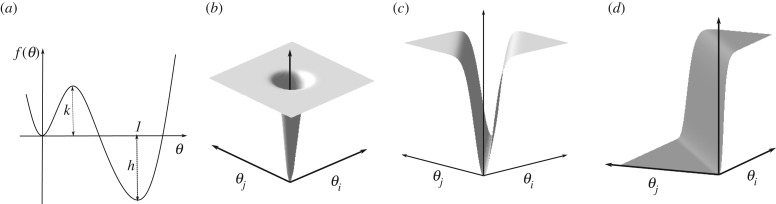
The individual energy terms for intra-protomer, intra-pentamer, inter-pentamer interactions and the terms for the mechanical and steric interactions. (*a*) Bistable intra-protomer energy *f*(*θ*); (*b*) plot of the function *g*(*θ*_*i*_, *θ*_*j*_) in the intra-pentamer and inter-pentamer interaction energies; (*c*) plot of the function *r*(*θ*_*i*_, *θ*_*j*_) accounting for the steric and mechanical interactions around a threefold axis; (*d*) plot of the function *s*(*θ*_*i*_, *θ*_*j*_) accounting for the steric and mechanical interactions around a fivefold axis. (Online version in colour.)

Let henceforth *f*(*θ*) be a two-well energy as in figure 3*a*, parametrized by two coefficients *k* and *h*, such that

— *f* has two minima, one at *θ* = 0 with *f*(0) = 0 and the other at *θ* = 1, that correspond to the closed and open configurations of the protomer (after the pocket factor has been removed), respectively; the quantity *f*(1) = *h* < 0 is the depth of the energy well corresponding to the open configuration of the protomer.
— *f* has a single maximum at *θ*_*m*_ ∈ (0, 1), whose value is *f*(*θ*_*m*_) = *k* > 0. Thus *k* represents the energy barrier between the wells and reflects the number of bonds that must be broken in order to complete the rotation of the VP1 subunits. According to table 1, the relevant proteins interacting are (see also figure 1):
  2.1 VP1a:VP2a, VP1a:VP3a, VP2a:VP3a.

We assume that the total intra-protomer energy of the capsid has the form

2.2 $$E_{\mathrm{protomer}}(\theta )=\sum_{i=1}^{60}f(\theta_{i}).$$

In the simulations described later, we have tested four different explicit forms for the function *f*, without finding any significant difference. The simplest one is the fourth-order polynomial

$$f(x)=hx^{2}\frac{\left(3x^{2}-4(p+1)x+6p\right)}{\left(2p-1\right)},$$

with *p* a solution of *p*^3^(*p* − 2)/(2*p* − 1) = −*k*/*h*, where *k* and *h* are as in figure 3*a*. We have also tried piecewise smooth functions obtained by interpolating fixed points with splines and, finally, a Gaussian mixture model

$$f(x)=-\ln(a_{1}\;{\text{e}}^{-(x^{2}/s_{1})}+a_{2}\;{\text{e}}^{-({\left(x-1\right)}^{2}/s_{2})}),$$

with *a*_1_ = 1, *a*_2_ = 400, *s*_1_ = 1/20, *s*_2_ = 1/15, inspired by recent techniques developed to reconstruct low-dimensional dynamical transition networks from high-dimensional static samples [29].

### 2.3. Intra-pentamer interactions

This term refers to the cohesive interactions among two adjacent protomers in the same pentamer. Specifically, according to table 1, the intra-pentamer interactions are

2.3 $$\begin{array}{rl} & VP1a:VP1b,\;VP1a:VP3b,\;VP2a:VP1b\\ & VP2a:VP3b,\;VP3a:VP1b,\;VP3a:VP3b, \end{array}$$

that, referring to the Schlegel diagram in figure 2*a*, correspond to the red segments in figure 2*b*.

The corresponding intra-pentamer energy is the energy that is required in order to break the intra-pentamer bonds that block the opening of each protomer. We assume it to have the general form

2.4 $$E_{\mathrm{pentamer}}(\theta )=\frac{1}{2}\sum_{i,j=1}^{60}A_{ij}g(\theta_{i},\theta_{\;j}),$$

where *A*_*ij*_ is the adjacency matrix of the graph in figure 2*b*, whose nodes are the protomers and whose edges are the intra-pentamer bonds (red segments). We require that the function *g* has the form $g(\theta_{i},\theta_{\;j})=\tilde{g}(a(\theta_{i}^{2}+\theta_{\;j}^{2}))$ where $\tilde{g}$ is smooth and monotonically increasing from 0 to 1 in the interval [0, + ∞), $\tilde{g}(0)=0$, and $\tilde{g}(x)\equiv 1$ for *x* ≥ 1; here 1/*a* > 0 is a measure of a typical interaction radius. In our simulations, we will use

2.5 $$g(\theta_{i},\theta_{\;j})=1-\;{\text{e}}^{-a(\theta_{i}^{2}+\theta_{\;j}^{2})},$$

with *a* ≫ 1 sufficiently large (cf. also [30] and figure 3*b*).

The interaction energy *g*(*θ*_*i*_, *θ*_*j*_) has a sharp minimum at *θ*_*i*_ = *θ*_*j*_ = 0, i.e. when both adjacent protomers are closed and undeformed. However, when either *θ*_*i*_ = 1 or *θ*_*j*_ = 1, so that at least one of the protomers is open, the energy is maximal, since it is sufficient that one of the protomers is open to break the bonds involved in the interaction with its neighbours.

### 2.4. Inter-pentamer interactions

This term accounts for the contributions of the cohesive interactions among protomers belonging to different pentamers, that we identify with the energy of the bonds between these protomers that have to be disrupted in order to expand the capsid. We distinguish between interactions around the threefold and twofold axes. According to table 1, the inter-pentamer interactions around the threefold axes are

2.6 VP1a:VP2g, VP3a:VP2g, VP3a:VP3g.

Referring to the Schlegel diagram in figure 2*a*, the inter-pentamer interactions around the threefold axes are indicated by the blue segments in figure 2*d*. Analogously, the interactions across twofold axes, i.e.

2.7 VP2a:VP2m,

correspond to the green segments in figure 2*d*. Consistent with this, we assume that the inter-pentamer energy is the sum of two contributions,

2.8 $$\begin{array}{rl} & E_{3\mathrm{fold}}(\theta )=\frac{1}{2}\sum_{i,j=1}^{60}B_{ij}g(\theta_{i},\theta_{\;j})\;\text{and}\;\\ & \;E_{2\mathrm{fold}}(\theta )=\frac{1}{2}\sum_{i,j=1}^{60}C_{ij}g(\theta_{i},\theta_{\;j}), \end{array}$$

where *B*_*ij*_ and *C*_*ij*_ are the adjacency matrices of the graphs in figures 2*c*,*d* respectively, i.e. the graphs whose nodes are the protomers and whose edges are the intra-pentamer bonds, and the function *g* is as in (2.5).

### 2.5. Mechanical constraints

Neighbouring proteins tend to impact on each others motion via steric and mechanical constraints. To simplify, we assume that protomers push and pull each other across interfaces within the pentamer (figure 2*b*/ fivefold axes), and interfaces between different pentamers (figure 2*d*/ threefold axes). Because of the relative positions of the VP1 domains involved in the openings of the individual protomers, we only need to consider interactions at the *ab* (cf. (2.3)) and *ag* interfaces (cf. (2.6)), neglecting possible steric interactions across the *am* interface (VP2a:VP2m). This is because the interface lies at the twofold axis where the main pore opens as protomer domains move away from each other; in fact, the expansion of the capsid tends to separate these interfaces, after breaking the cohesive bonds involved in the inter-protomer energy.

We account for the steric and mechanical interactions by energetic terms that penalize the separation between adjacent protomers. In our simplified model, we do not study the actual motion of the protomers in detail, but we assume that it can be represented by a function of the variables *θ*_*i*_, so that it is reasonable to enforce the constraints by penalizing the difference *θ*_*i*_ − *θ*_*j*_, where *i* and *j* are adjacent protomers.

To describe the steric and mechanical interactions between different pentamers around the threefold axes, we penalize the relative motion of the protomers through a term of the form

2.9 $$E_{S3}(\theta )=\frac{1}{2}\sum_{ij=1}^{60}C_{ij}r(\theta_{i},\theta_{\;j}),$$

where *C*_*ij*_ is the adjacency matrix of the graph in figure 2*d* and moreover $r(\theta_{i},\theta_{\;j})=\tilde{r}(b{\left(\theta_{i}-\theta_{\;j}\right)}^{2})$ (cf. figure 3*c*). Here, the function $\tilde{r}(x)$ satisfies the same requirements as the function $\tilde{g}$ in §2.3, and *b* ≫ 1 is a real constant measuring the rigidity of the proteins, with small values meaning soft and large values meaning rigid. In our simulations, we shall use

2.10 $$r(\theta_{i},\theta_{\;j})=1-\;{\text{e}}^{-b{\left(\theta_{i}-\theta_{\;j}\right)}^{2}}.$$

As to the intra-pentamer interfaces, since the opening of a protomer induces a clockwise rotation of the pentamers, we can assume that the opening results in a push of the adjacent clockwise protomer. This introduces a chirality in the corresponding penalization of the energy, that has the form

2.11 $$E_{S5}(\theta )=\sum_{ij=1}^{60}\;\;{\tilde{A}}_{ij}s(\theta_{i},\theta_{\;j}),$$

where

$$\;\;{\tilde{A}}_{ij}=\left\{ \begin{array}{cc} A_{ij}\; & \mathrm{if}\;i>j\;\mathrm{mod}\;5\;\\ 0\; & \mathrm{otherwise}\;\; \end{array}\right.$$

is the matrix that takes into account only the clockwise adjacencies within the pentamer, and the push force from *i* to *j* is accounted for by the function

$$s(\theta_{i},\theta_{\;j})=\left\{ \begin{array}{cc} r(\theta_{i},\theta_{\;j})\; & \text{if}\;\theta_{i}>\theta_{\;j},\;\\ 0\; & \text{otherwise.}\; \end{array}\right.$$

### 2.6. The total energy

In summary, the total energy is

2.12 $$\begin{array}{rl} E(\theta ) & =E_{\mathrm{protomer}}(\theta )+c_{1}E_{\mathrm{pentamer}}(\theta )+c_{2}E_{2\mathrm{fold}}(\theta )\\ & \;+c_{3}E_{3\mathrm{fold}}(\theta )+c_{4}E_{S3}(\theta )+c_{5}E_{S5}(\theta ), \end{array}$$

where *c*_*i*_, *i* = 1 … 5 are parameters indicating the relative strengths of individual energy contributions. In particular, the functions *E*_pentamer_, *E*_2fold_, *E*_3fold_, *E*_S3_ and *E*_S5_ are all normalized to the same height of the plateau, so that the energy barriers corresponding to the number of bonds lost in the rearrangement of the capsid across the interfaces (2.3), (2.6), and (2.7), respectively, are accounted for by the parameters *c*_1_, *c*_2_ and *c*_3_.

Recall now that the intra-protomer energy *E*_protomer_ involves two parameters *k* and *h*, with *k* the energy barrier necessary to break the bonds within the protomer and allow for its opening (cf. figure 3*a*). The actual value of the barrier is 89.5 kcal mol^−1^ (cf. table 1), and corresponds to the number of bonds broken during the transition according to table 1. We normalize the energy so that *k* = 1 by dividing all constants by this value, i.e.

2.13 $$c_{1}=\frac{92.8}{89.5},\;c_{2}=\frac{24.6}{89.5}\;\text{and}\;c_{3}=\frac{48.9}{89.5}.$$

The constants *c*_4_ and *c*_5_ represent values of the energy required to violate the steric constraints. In the absence of experimental values, we will discuss below their role and the impact of different choices for these values. As to the constant *h*, which is the depth of the well of the intra-protomer energy corresponding to the open state of the protomer, we have chosen it to be *h* = −6 in order to ensure that the total energy decreases at each transition event (cf. §3.1).

## 3. The expansion of the capsid

Our model describes the expansion of the capsid via the sequential opening of one or more protomers as a sequence of transitions between metastable states, i.e. local minima, of the total energy. To explore the energy landscape of the capsid we use a technique borrowed from the theory of large deviations [31], based on the assumption that the transitions occur along minimum energy paths (MEPs), which are paths connecting minima that occur with maximum probability when small stochastic fluctuations are included into the model [32].

### 3.1. Minimum energy paths

We briefly review below the above technique, referring to [32–35] for a detailed motivation and description.

To describe MEPs between two minima of the energy function, assume that small fluctuations around a minimum are described by a stochastic dynamical system of the form $d\theta =-\nabla_{\theta }E\;\text{d}t+\epsilon \;\text{d}W$, where *ε* tends to zero (*ε* ≪ 1) and ***W*** is a multidimensional Brownian motion. Every trajectory of this system starting near a minimum exits its basin of attraction with probability 1 and enters the basin of attraction of another minimum, and the trajectories concentrate with high probability near a special path that connects the two minima, called the MEP. The MEP between two minima can be computed by a technique invoking a curve evolution equation [32–34].

Consider now the collection of all metastable states of the capsid, called the state space S. The cardinality of S is 2^60^, implying that the problem of studying all possible transitions is computationally intractable. Therefore, we have restricted our study only to transitions that involve the opening of a single protomer at a time. We have also numerically explored some reverse transitions, corresponding to the closing of some of the protomers, as well as some of those involving the simultaneous opening of at least two protomers, but we have consistently found larger energy barriers than those corresponding to single protomer openings.

Hence, we define a discrete-time Markov chain with state space S such that the transition probability from state ***θ*** to state ***θ*** ′ is

3.1 $$P(\theta ,\theta^{\prime })=\frac{\exp(-2\beta (\theta ,\theta^{\prime })/\epsilon^{2})}{\sum_{\;\;\tilde{\theta }\in S}\exp(-2\beta (\theta ,\;\tilde{\theta })/\epsilon^{2})},$$

where the states ***θ*** and ***θ***′ are such that there exists *i* for which *θ*_*i*_ = 0 and *θ*′_*i*_ = 1, and *θ*_*j*_ = *θ*′_*j*_ ∈ {0, 1} for *j* ≠ *i*, and there is a MEP between ***θ*** and ***θ*** ′ such that the energy computed along the path has a single maximum at a point ***θ*** *. We set *P*(***θ***, ***θ*** ′) = 0 otherwise. If the transition probability is not zero, we define the barrier between the two states as [35]

3.2 $$\beta (\theta ,\theta {\;}^{\prime })=E(\theta^{\;*})-E(\theta ).$$

Note that our choice of the transition probabilities implies that we are forcing the transition to be irreversible, which is not usually the case in large deviations theory. Our choice follows from the fact that closed empty HRV2 capsids have not been observed experimentally, which suggests that the actual process of expansion is indeed not reversible, even though there are some viruses, such as ERAV, for which closed empty capsids are observed *in vitro* under specific experimental conditions.

Irreversibility is also implied by the fact that the barriers for the forward transitions (closed to open) in our model are consistently smaller than those for the reverse transition. This is indeed a consequence of our choice of the intra-protomer energy *f*. In fact, according to our general scheme, the expansion of the capsid is due to the protomer reaching a more stable position, when the obstruction due to the pocket factor is removed. This is modelled by the deep well in the function *f*(*θ*). All other energy terms are cohesive and counteract the expansion. The likelihood of the reverse process (i.e. closing versus opening) is related to the relative depths of the energy wells: if the ‘open’ well is deeper than the ‘closed’ well, it is less probable to go back to the closed state than to go forward from the closed to the open state. Hence, in order to account for the difficulty (if not impossibility) of the reverse transition, we have assumed the energy well in the intra-protomer energy *f* to be deep enough so that the global energy (the sum of all contributions) actually decreases in each protomer opening, and the barrier for closing is higher than the barrier for opening. Note finally that this fact also implies that the energy decreases at each transition step: the energy cascade.

### 3.2. An approximate expression of the barriers

We prove in appendix A three basic facts that facilitate the analysis of the transition mechanism. In particular, for large *a* and *b*, we have the following results:

(a) The configurations in which each protomer is either closed (*θ*_*i*_ ∼ 0) or open (*θ*_*i*_ ∼ 1) are local minima of the energy.
(b) The MEPS are approximately rectilinear paths between minima: in particular, focusing on transitions that correspond to the opening of a single protomer, let ***θ*** and ***θ*** ′ be two metastable states such that *θ*_*j*_ = *θ* ′_*j*_ if *j* ≠ *i* and *θ*_*i*_ = 0, *θ*_*i*_ ′ = 1 for some protomer *i*. Then, if a MEP connecting ***θ*** and ***θ*** ′ exists, its parametric expression as *b* → +∞ is
  3.3 $$\theta_{i}(t)∼t,\;\theta_{\;j}(t)∼\theta_{\;j}\equiv \theta {\;}_{\;j}^{\prime }\;\text{for}\;j\neq i,\;t\in [0,1].$$
(c) Finally, the barriers can be estimated in terms of the constants *k* and *c*_1_, …, *c*_5_ in the expression of the total energy (2.12). On MEP (3.3), corresponding to the opening of protomer *i*, the barriers are
  3.4 $$\begin{array}{rl} \beta (\theta ,\theta {\;}^{\prime })∼k & +\sum_{j/\theta_{\;j}=0}(c_{1}A_{ij}+c_{2}B_{ij}+c_{3}C_{ij})\\ & \;+\sum_{j/\theta_{\;j}=0}(c_{4}C_{ij}+c_{5}\;{\tilde{A}}_{ij}). \end{array}$$

## 4. Results

### 4.1. Transition pathways

We first address the issue of whether the expansion of the capsid is likely to occur either by a sequential process of successive openings of adjacent protomers, or by a disordered generalized opening of many or all protomers at different unrelated sites.

To assess the likely transition mechanism, we note that the barrier to opening a single protomer in the native configuration of the capsid according to (3.4) is

4.1 $$\beta (\theta_{0},\theta_{1})=k+2c_{1}+c_{2}+2c_{3}+2c_{4}+c_{5},$$

where ***θ***_0_ = (0, 0, …, 0) and, without loss of generality due to the symmetry of the capsid, ***θ***_1_ = (1, 0, …, 0). Now consider the second transition event: if the protomer that opens is not adjacent to protomer *i* = 1, the energy barrier is still (4.1); otherwise, if it is adjacent to 1, then the barrier is smaller than (4.1) (see below). Hence, the most probable transition path is a sequence of openings of adjacent protomers initiated at a single nucleation site, rather than a sequence of random openings at unrelated protomers.

Next, we study how the initial stages of the expansion depend on the parameters *b*, *c*_4_, and *c*_5_ in the mechanical constraint energy. Here we interpret *b* as playing the role of an elasticity modulus, with larger *b* meaning more rigid proteins, and *c*_4_, *c*_5_ indicating the strength of the mechanical constraints on proximal protomers.

**Figure 4.**
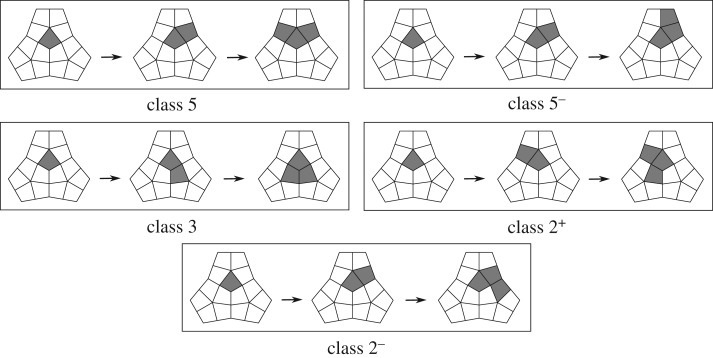
Classes of transition pathways. Different transition pathways are classified according to the positions of the second and the third protomer undergoing an opening event.

Numerical simulations (cf. table 2) and the argument presented in appendix A show that the initial stages of the transition belong to a small number of different classes, depicted in figure 4 and labelled according to their positions relative to five-, three- and twofold axes of the particle:

— **Class** 5: After the opening of the first protomer, the transition proceeds by opening adjacent protomers in the same pentamer, either clockwise or anti-clockwise around the fivefold axis. This occurs when *c*_5_ = 0 and *c*_4_ is small, so that the mechanical constraint around the threefold axis is weak and there is no chiral constraint around the fivefold axes.
— **Class** 5^**−**^: The transition proceeds by opening adjacent protomers belonging in the same pentamer, in counter-clockwise order. This occurs when the chiral intra-pentamer constant *c*_5_ is large and much larger than *c*_4_.
— **Class** 3: The transition proceeds by opening adjacent protomers in the same trimer, either clockwise or anti-clockwise around the threefold axis. This occurs when the constraint around the threefold axis is strong, i.e. when the constant *c*_4_ is large and much larger than *c*_5_.
— **Classes** 2^–^, 2^+^: After the opening of the first protomer, the transition proceeds by opening its neighbour in the same pentamer, either in clockwise (+) or counter-clockwise (−) order, according to whether *c*_5_ = 0 or not. The third protomer to open is then the one located across the twofold axis of the dimer, and this leads to the opening of a pore at this twofold axis. Using the values (2.13) for *c*_1_, *c*_2_, *c*_3_, this happens when the constraints have comparable strength, i.e. when 0.21 < *c*_4_ − *c*_5_ < 0.49.

**Table 2.** Results of numerical simulations showing the initial modes of transition for *a* = *b* = 100, classified according to the classes in figure 4 for different choices of *c*_4_ (rows) and *c*_5_ (columns).

	*c*_4_																				
*c*_5_	0	0.1	0.2	0.3	0.4	0.5	0.6	0.7	0.8	0.9	1	1.1	1.2	1.3	1.4	1.5	1.6	1.7	1.8	1.9	2
0	5	5 −	5 −	5 −	5 −	5 −	5 −	5 −	5 −	5 −	5 −	5 −	5 −	5 −	5 −	5 −	5 −	5 −	5 −	5 −	5 −
0.1	5	5 −	5 −	5 −	5 −	5 −	5 −	5 −	5 −	5 −	5 −	5 −	5 −	5 −	5 −	5 −	5 −	5 −	5 −	5 −	5 −
0.2	2+	5 −	5 −	5 −	5 −	5 −	5 −	5 −	5 −	5 −	5 −	5 −	5 −	5 −	5 −	5 −	5 −	5 −	5 −	5 −	5 −
0.3	2+,2 −	2 −	5 −	5 −	5 −	5 −	5 −	5 −	5 −	5 −	5 −	5 −	5 −	5 −	5 −	5 −	5 −	5 −	5 −	5 −	5 −
0.4	3,2 −	2 −	2 −,5 −	5 −	5 −	5 −	5 −	5 −	5 −	5 −	5 −	5 −	5 −	5 −	5 −	5 −	5 −	5 −	5 −	5 −	5 −
0.5	3	3,2 −	2 −	2 −	5 −	5 −	5 −	5 −	5 −	5 −	5 −	5 −	5 −	5 −	5 −	5 −	5 −	5 −	5 −	5 −	5 −
0.6	3	3	3	2 −	2 −	5 −	5 −	5 −	5 −	5 −	5 −	5 −	5 −	5 −	5 −	5 −	5 −	5 −	5 −	5 −	5 −
0.7	3	3	3	3	2 −	2 −	5 −	5 −	5 −	5 −	5 −	5 −	5 −	5 −	5 −	5 −	5 −	5 −	5 −	5 −	5 −
0.8	3	3	3	3	3,2 −	2 −	2 −	5 −	5 −	5 −	5 −	5 −	5 −	5 −	5 −	5 −	5 −	5 −	5 −	5 −	5 −
0.9	3	3	3	3	3	3,2 −	2 −	2 −	5 −	5 −	5 −	5 −	5 −	5 −	5 −	5 −	5 −	5 −	5 −	5 −	5 −
1	3	3	3	3	3	3	3,2 −	2 −	2 −	5 −	5 −	5 −	5 −	5 −	5 −	5 −	5 −	5 −	5 −	5 −	5 −
1.1	3	3	3	3	3	3	3	3,2 −	2 −	2 −	5 −	5 −	5 −	5 −	5 −	5 −	5 −	5 −	5 −	5 −	5 −
1.2	3	3	3	3	3	3	3	3	3,2 −	2 −	2 −,5 −	5 −	5 −	5 −	5 −	5 −	5 −	5 −	5 −	5 −	5 −
1.3	3	3	3	3	3	3	3	3	3	3	2 −	2 −	5 −	5 −	5 −	5 −	5 −	5 −	5 −	5 −	5 −
1.4	3	3	3	3	3	3	3	3	3	3	3	2 −	2 −	5 −	5 −	5 −	5 −	5 −	5 −	5 −	5 −
1.5	3	3	3	3	3	3	3	3	3	3	3	3	2 −	2 −	5 −	5 −	5 −	5 −	5 −	5 −	5 −
1.6	3	3	3	3	3	3	3	3	3	3	3	3	3,2 −	2 −	2 −	5 −	5 −	5 −	5 −	5 −	5 −
1.7	3	3	3	3	3	3	3	3	3	3	3	3	3	3,2 −	2 −	2 −	5 −	5 −	5 −	5 −	5 −
1.8	3	3	3	3	3	3	3	3	3	3	3	3	3	3	3,2 −	2 −	2 −	5 −	5 −	5 −	5 −
1.9	3	3	3	3	3	3	3	3	3	3	3	3	3	3	3	3,2 −	2 −	2 −	5 −	5 −	5 −
2	3	3	3	3	3	3	3	3	3	3	3	3	3	3	3	3	3,2 −	2 −	2 −	5 −	5 −

We now show how the above classification can also be derived from (3.4). Assume, without loss of generality, that protomer 1 has opened, and consider now the second opening event. The protomers that are adjacent to *i* = 1 are 2, 5, 6, 10, 30 (figure 2*a*), and the corresponding energy barriers are

$$\begin{array}{rl} & \beta (\theta_{1},\theta_{1,2})=k+c_{1}+c_{2}+2c_{3}+2c_{4},\\ & \beta (\theta_{1},\theta_{1,5})=k+c_{1}+c_{2}+2c_{3}+2c_{4}+c_{5},\\ & \beta (\theta_{1},\theta_{1,6})=\beta (\theta_{1},\theta_{1,30})=k+2c_{1}+c_{2}+c_{3}+c_{4}+c_{5}\\ \text{and}\; & \beta (\theta_{1},\theta_{1,10})=k+2c_{1}+2c_{3}+2c_{4}+c_{5}, \end{array}\;$$

where we have denoted by ***θ***_*a*,*b*,…,*c*_ the state in which *θ*_*a*_ = *θ*_*b*_ = … = *θ*_*c*_ = 1, and *θ*_*j*_ = 0 for all *j* ≠ *a*, *b*, …, *c*. Therefore,

$$\begin{array}{rl} & \beta (\theta_{1},\theta_{1,2})<\beta (\theta_{1},\theta_{1,5})\Leftrightarrow 0<c_{5},\\ & \beta (\theta_{1},\theta_{1,2})<\beta (\theta_{1},\theta_{1,6})=\beta (\theta_{1},\theta_{1,30})\Leftrightarrow c_{3}+c_{4}<c_{1}+c_{5}\\ \text{and}\; & \beta (\theta_{1},\theta_{1,2})<\beta (\theta_{1},\theta_{1,10})\Leftrightarrow c_{2}<c_{1}+c_{5}\;\\ & \;\;\;\;\;\;(\text{always true since}\;c_{2}<c_{1}). \end{array}$$

The first observation is that, when *c*_5_ > 0, the opening of protomer 2, adjacent to 1 in the counter-clockwise direction, is preferred to the opening of the protomer 5, adjacent to 1 in the clockwise position. Hence, the classes 5 and 2^+^ can only be realized when *c*_5_ = 0. If also *c*_4_ is small, the sequence of the openings is then determined just by the competition between the bond energies *c*_1_, *c*_2_ and *c*_3_. Since *c*_1_ > *c*_2_, *c*_3_ the energy decrease due to the disruption of the intra-pentamer interface 1–2 or 1–5 dominates and the barrier for the opening of 2 or 5 is lower than all other barriers.

However, when *c*_5_ > 0, the intra-pentamer penalization term (2.11) is such that the opening of protomer 1 generates a push on the clockwise adjacent protomer 5 (when this is in the closed state) that costs energy. If protomer 5 is in the open state, there is no push and no corresponding energy penalization. Hence, assume that 1 is open. Then by the above argument the opening of protomer 2 would not generate a push on its clockwise neighbour 1 and would not require energy. On the other hand, the opening of 5 would generate a push on protomer 4 (figure 2*a*), which is closed, and this would require to overcome an energy barrier corresponding to *c*_5_. Hence, even though the opening of the protomers generates a clockwise push, the sequence by which they open propagates counter-clockwise, as in rarefaction waves.

Actually, the opening of protomer 5 also should release the energy accumulated in the push from protomer 1 at its counter-clockwise side, so that the net gain of energy should be zero, as for the opening of protomer 2. However, by our assumptions on the form of the chiral constraint energies, the relaxation of the constraint is felt only when the opening of protomer 5 is almost complete, while the expenditure of energy due to the push on 4 operates already at the first stages of the opening, so that it does represent a barrier to overcome to initiate the process (cf. expression (A 4)).

A stronger statement is that, if the steric constraint energy thresholds *c*_4_ and *c*_5_ are such that

4.2 $$c_{4}-c_{5}<c_{1}-c_{3}\;\text{and}\;c_{5}>0,$$

then, with highest probability, the second protomer to open is necessarily protomer 2 (recall that *c*_3_ < *c*_1_ by (2.13)).

Assume now that the second protomer that opens is protomer 2, and compute the barriers required to open any of the protomers adjacent to the pair 1–2, i.e. 3, 6, 10, 11, 15, 30. We get

$$\begin{array}{rl} & \beta (\theta_{1,2},\theta_{1,2,10})=k+2c_{1}+c_{3}+c_{4}+c_{5},\\ & \beta (\theta_{1,2},\theta_{1,2,3})=k+c_{1}+c_{2}+2c_{3}+2c_{4},\\ & \beta (\theta_{1,2},\theta_{1,2,15})=k+2c_{1}+2c_{3}+2c_{4}+c_{5}\\ \text{and}\; & \beta (\theta_{1,2},\theta_{1,2,11})=\beta (\theta_{1,2},\theta_{1,2,6})=\beta (\theta_{1,2},\theta_{1,2,30})\\ & \;\;\;\;\;=k+2c_{1}+c_{2}+c_{3}+c_{4}+c_{5}. \end{array}\;$$

Therefore, we have first that always

$$\begin{array}{rl} & \beta (\theta_{1},\theta_{1,2,10})<\beta (\theta_{1},\theta_{1,2,15}),\\ & \;\beta (\theta_{1},\theta_{1,2,10})<\beta (\theta_{1},\theta_{1,2,11})=\beta (\theta_{1},\theta_{1,2,6})=\beta (\theta_{1},\theta_{1,2,30}), \end{array}$$

and

$$\begin{array}{rl} & \beta (\theta_{1},\theta_{1,2,10})<\beta (\theta_{1},\theta_{1,2,3})\Leftrightarrow c_{1}+c_{5}<c_{2}+c_{3}+c_{4},\\ & \beta (\theta_{1},\theta_{1,2,3})<\beta (\theta_{1},\theta_{1,2,11})\Leftrightarrow c_{3}+c_{4}<c_{1}+c_{5}\\ \text{and}\; & \beta (\theta_{1},\theta_{1,2,3})<\beta (\theta_{1},\theta_{1,2,15})\Leftrightarrow c_{2}<c_{1}+c_{5}\;\\ & \;\;\;\;\;(\text{always true since}\;c_{2}<c_{1}). \end{array}\;$$

Hence, if

4.3 $$0<c_{1}-c_{2}-c_{3}<c_{4}-c_{5}<c_{1}-c_{3},$$

the third protomer that opens is protomer 10, with highest probability, and we recover class 2^−^, which requires the interplay of both mechanical constraints to be operative.

Analogously, always in the hypothesis that the second protomer to open is 2, the opening proceeds along the pentamer in a counter-clockwise direction and belongs to class 5^−^ when

4.4 $$c_{4}-c_{5}<c_{1}-c_{2}-c_{3},$$

since in this case *c*_4_ is too small to force the opening along a trimer.

Finally, a similar analysis shows that the transition proceeds by opening a full trimer, i.e. it belongs to class 3, if

4.5 $$c_{1}-c_{3}<c_{4}-c_{5}\;\text{and}\;c_{1}+c_{2}-c_{3}<c_{4}.$$

Hence, as expected, the competition between the steric constraint energies determines the kinetics of the opening sequence of the protomers.

### 4.2. The pores at the twofold axes

As mentioned in §2, during the expansion a number of pores open in the capsid: the pores at the twofold axes seem to be the most important, because they are the widest, and allow both for the VP4 and the RNA to exit from the capsid. The opening of these pores is due to the clockwise rotation of two facing protomers, for instance those labelled *a* and *m* in figure 1*a*. In particular, the pore is formed when VP2a moves away from VP2m.

In our schematics in figure 4 showing the first three steps of the transition pathways, this only occurs at the third stage of classes 2^+^ and 2^−^. In all other transition paths, this generally occurs at a later stage.

Furthermore, in classes 2^+^ and 2^−^, the position of the first pore to open is uniquely determined, while in the other expansion modes the location of the first twofold pore to open is random.

## 5. Discussion

Viral capsids are metastable structures—stable enough to provide protection for the viral genome, yet able to release their genomic cargo upon environmental cues. An important feature regulating this balance is the cooperative effects of multiple, relatively weak interactions between neighbouring coat proteins.

**Figure 5.**
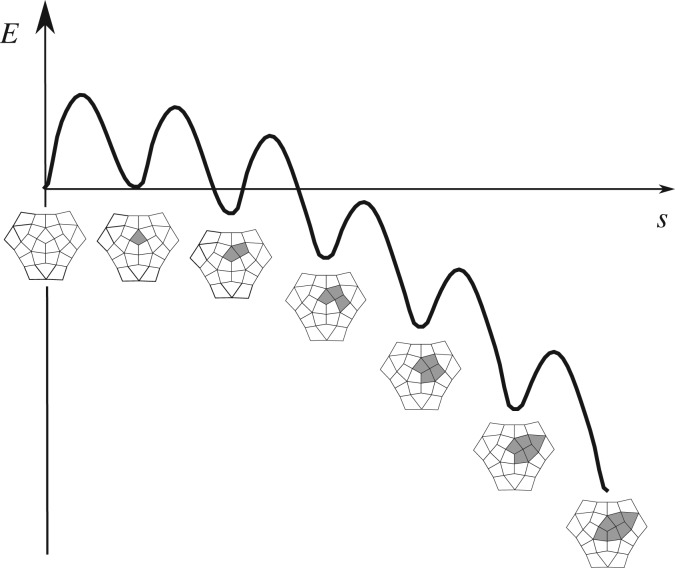
A typical energy cascade for the opening of the first 6 protomers in mode 2^−^. The energy is plotted along a path in $R^{60}$ that is the union of the first 6 MEPs, and *s* is the arc parameter along the path.

Previous studies demonstrate that the most energetically favourable mechanism for the global conformational change of the capsid during infection is a sequence of elementary conformational changes of single or small groups of protomers, that weaken the cooperative effect of the cohesive energies and destabilize adjacent protomers [28]. At each stage, the total energy decreases, as well as the threshold for each individual transition (figure 5). This mechanism has been experimentally observed in the maturation of HK97 in [26,27], and studied on simple capsid models in [17,28]. Each elementary change is viewed as a path between two energy minima on the energy landscape, and the precise sequence of openings can be determined by exploring the energy landscape via MEPs.

In this work, we have applied the above approach to HRV2, with the purpose of contributing some working hypotheses to the research on the infection mechanism of HRV2, and improve the understanding of the factors that promote the release of the genome in the host cell.

Specifically, for this type of virus, no receptor is involved in the externalization of the RNA and its inclusion into the host cell. Once the virus is internalized into the early endosome, the change of pH within the endosome induces the structural modifications leading to the expansion of the capsid described in §2. As discussed there, as a result of the expansion, pores open at twofold axes of the capsid, the N-terminal arm of VP1 is externalized and VP4 exits the capsid: the N-terminal arm of VP1 is hydrophobic and is thought to be important in the adhesion of the capsid to the interior of the endosomal membrane.

Furthermore, the RNA exit from the capsid starts by extruding the 3^ ′^-end from a pore at one of the twofold axes [18,24,25].

Experimental evidence shows that the endosome is not disrupted during the exit of the RNA into the cell [36]. Hence, it is likely that the RNA exits from the endosome into the cell through pores in the endosomal membrane, and it seems that VP4 is instrumental in the formation of these pores.

A first hypothesis is that, in the absence of any regulatory mechanism, the capsid expands and all pores at the particle twofold axes open simultaneously. In this case, the 3’-end of the RNA is free to exit the capsid via any available pore (most likely the one which is nearest to its location in the interior of the capsid), but it faces the problem of exiting the endosome and entering the cell through one of the pores in the endosomal membrane. There are two possible ways to achieve this: either the RNA is extruded into the endosome and must then find a pore (which is an unlikely scenario as the genome would be degraded in the endosome), or the pore in the capsid is directly adjacent to a pore in the endosomal membrane. However, if the pore through which the RNA exits is not defined, any pores at any of the 30 particle twofold axes could contact the endosomal membrane, making this process inefficient and thus unlikely.

This suggests the following alternative. If the expansion is initiated at the site at which the 3^ ′^-end of the RNA is located, and if the expansion pathway is such that it preferentially opens the pore nearest to the 3^ ′^-end, then VP4 would be externalized at that capsid pore first, enabling it to open a pore in the endosomal membrane just facing this pore. This would then result in formation of a channel through which the RNA could exit both the capsid and the endosome and enter the cell. Then the expansion would proceed, but there would be no need for the opening of further pores in the endosomal membrane, and the problem of the localization and matching of the three players in this game (the 3^ ′^-end of the RNA, the pore in the capsid and the pore in the endosomal membrane) would be solved. Also, if the first twofold pore that opens during the transition pathway was uniquely determined in this way in proximity to the binding site at the 3^ ′^-end of the RNA, this would ensure that the RNA could exit even if capsid expansion was stopped or slowed down after a few steps.

We have shown here that a simple coarse-grained model can account for initial pore opening immediately near the 3^ ′^-end of the RNA, and that this automatically occurs for every energy-decreasing transition pathway, if the constants *c*_4_ and *c*_5_ satisfy reasonable constraints, i.e. *c*_4_ ∼ *c*_5_. This is highly likely, given that *c*_4_ and *c*_5_ are related to the mechanical response of two different interfaces of the same protein.

However, our coarse-grained model cannot explain how binding of the 3^ ′^-end of the RNA to a protomer may promote the initiation of the structural capsid transition at this protomer. There are precedent cases for RNA–CP contacts changing the conformation of capsomers and their ability to interact with other capsomers in the capsid shell, such as the allosteric conformers switch of the MS2 dimer triggered by contact with the RNA. Our model suggests that, similarly, the RNA–CP interactions at the protomer in contact with the 3^ ′^-end of the RNA might account for its role in initiating the transition pathway. However, this suggestion requires experimental validation.

In summary, our model is designed to demonstrate that it is possible, for a realistic range of the structural parameters describing the capsid, to envisage a simple mechanism by which the three fundamental players (the 3^ ′^-end of the RNA, the pore in the capsid and the pore in the endosomal membrane) act in a coordinated fashion without the need for further regulation or any indeterminacy to ensure an efficient and fast release of the genome into the host cell.

## Data accessibility

This article has no additional data.

## Appendix A. Metastable states, minimum energy paths and barriers

We provide here a justification for assertions (a), (b) and (c) in §3.2.

To prove (a), note that when *θ*_*i*_ ∈ {0, 1} for all *i*, each term in the total energy (2.12) either attains a local minimum or is locally constant. Therefore, since the minimum of a sum is greater than or equal to the sum of the minima, it follows that all ***θ*** ∈ {0, 1}^60^ are minimizers. Other minimizers could in principle exist, but they cannot be found analytically. We have therefore performed a set of global numerical searches (trust-region algorithm with Matlab Global Optimization Toolbox), which showed no evidence of other local minima.

Assertion (b) has been also validated by numerical simulations, but an analytical proof can be sketched using an asymptotic expansion as *b* → ∞ of the action functional based on the notion of *Γ*-convergence [37]. The argument is as follows: it is known that the MEP connecting two minima ***θ***_0_ and ***θ***_1_ is a minimizer of the geometric action functional (cf. [34])

A 1 $$\begin{array}{rl} J(\theta ) & =\int_{0}^{1}\{|\nabla E(\theta (s))∥\theta {\;}^{\prime }(s)|+\nabla E(\theta (s))\cdot \theta {\;}^{\prime }(s)\}\;\text{d}s\\ & =\int_{0}^{1}|\nabla E(\theta (s))∥\theta {\;}^{\prime }(s)|\;\text{d}s+E(\theta (1))-E(\theta (0)), \end{array}$$

among all smooth curves ***θ***(*s*) such that ***θ***(0) = ***θ***_0_ and ***θ***(1) = ***θ***_1_. Since the last term is independent of the path, the MEP is also a minimizer of the functional $I(\theta )=\int_{0}^{1}|\nabla E∥\theta {\;}^{\prime }|\;\text{d}s$.

For simplicity, we write b=n∈N, *E*_*n*_ in place of *E*, and split the energy as the sum of two terms, the first of which is independent of *n*:

$$\begin{array}{rl} & E_{n}=E_{0}+c_{4}E_{S3}+c_{5}E_{S5},\\ & \;E_{0}=E_{\mathrm{protomer}}+c_{1}E_{\mathrm{pentamer}}+c_{2}E_{2\mathrm{fold}}+c_{3}E_{3\mathrm{fold}}. \end{array}$$

Introducing the functionals

$$\begin{array}{rl} & I_{n}(\theta )=\int_{0}^{1}|\nabla E_{n}(\theta (s))∥\theta {\;}^{\prime }(s)|\;\text{d}s,\;{\tilde{I}}_{0}(\theta )=\int_{0}^{1}|\nabla E_{0}(\theta (s))∥\theta {\;}^{\prime }(s)|\;\text{d}s\\ & \text{and}\;\;\;I_{0}(\theta )={\tilde{I}}_{0}(\theta )+\Phi (\theta (0),\theta {\;}^{\prime }(0))+\Psi (\theta (1),\theta {\;}^{\prime }(1)), \end{array}$$

with *Φ* and *Ψ* depending only on the boundary values of ***θ*** and ***θ*** ′, we first note that the MEP relative to ${\tilde{I}}_{0}$ is a straight line segment. In fact, let ***θ***_0_ and ***θ***_1_ be two metastable states such that $\theta_{\;j}^{0}=\theta_{\;j}^{1}\in \{0,1\}$ if *j* ≠ *i* and $\theta_{i}^{0}=0$, $\theta_{i}^{1}=1$ for some protomer *i*. We claim that the minimizer ***θ***(*s*), *s* ∈ [0, 1], of ${\tilde{I}}_{0}$ connecting ***θ***_0_ to ***θ***_1_ is

A 2 $$\begin{array}{rl} & \theta_{i}(s)=s,\;\theta_{\;j}(s)\equiv \theta_{\;j}^{0}=\theta_{\;j}^{1}\in \{0,1\}\\ & \;\text{for}\;j\neq i,\;s\in [0,1]. \end{array}$$

To see this, we resort to the characterization of the MEPs in [34] as parametrized curves ***θ*** = ***θ***(*s*) with the property that $\nabla E_{0}(\theta (s))$ is parallel to ***θ*** ′(*s*) for *s* ∈ [0, 1]. For *j* ≠ *i*, we have that

A 3 $$\frac{\partial E_{0}}{\partial \theta_{j}}=f{\;}^{\prime }(\theta_{\;j})+2a\sum_{k}(c_{1}A_{\;jk}+c_{2}B_{\;jk}+c_{3}C_{\;jk})\theta_{\;j}\exp(-a(\theta_{\;j}^{2}+\theta_{k}^{2})),$$

and since *f* ′(0) = *f* ′(1) = 0 and $\theta_{\;j}\exp(-a(\theta_{\;j}^{2}+\theta_{k}^{2}))∼0$ whenever *θ*_*j*_ ∈ {0, 1}, this term vanishes on (A 2), which is therefore the MEP for ${\tilde{I}}_{0}$. This implies that also the minimizers of *I*_0_ are the straight-line segments (A 2): consider in fact a putative minimizer of *I*_0_ that is not a straight line segment, and modify it on the interval (*δ*/2, 1 − *δ*/2) by a smooth curve that coincides with (A 2) on (*δ*, 1 − *δ*), so that the boundary values of ***θ*** and ***θ*** ′ do not change. Since (A 2) is a minimizer of ${\tilde{I}}_{0}$, as *δ* → 0 this would decrease *I*_0_, which is a contradiction.

We now turn to the asymptotics of the action functional. We say that *I*_*n*_ *Γ*-converges to *I*_0_ if (cf. [37])

*g*_1_: for every converging sequence ***θ***^*n*^ → ***θ*** (in a sense to be specified below) then $I_{0}(\theta )\leq \underset{n}{lim inf}I_{n}(\theta^{\;n})$;
*g*_2_: for every ***θ*** there exists a sequence ***θ***^*n*^ → ***θ*** such that *I*_0_(***θ***) = lim_*n*_ *I*_*n*_(***θ***^*n*^).

This notion of convergence for the action functional has the advantage that, if a sequence of minimizers of *I*_*n*_ converges to some ***θ***, then this is a minimizer of *I*_0_ (cf. [37]). In terms of the MEPs, this means that we can approximate the MEP for large *b* in terms of the minimizer of the action functional *I*_0_, which is a rectilinear path.

The choice of the topology that defines the convergence of the sequences ***θ***^*n*^ must guarantee that sequences of minimizers converge, and, at the same time, that *g*_1_ and *g*_2_ hold. To simplify matters, we choose here the $C^{1}([0,1],R^{60})$ norm, which is admittedly too strong to ensure the convergence of minimizers, but makes *g*_1_ and *g*_2_ simpler to prove. With some additional technical effort the result below can be extended to weak convergence in $W^{1,\infty }([0,1],R^{60})$, that guarantees the convergence of minimizers.

We sketch here a proof of the fact that *I*_*n*_ *Γ*-converges to *I*_0_ for a simplified problem in two dimensions, with ***θ*** = (*θ*_1_, *θ*_2_), assuming that the energy reduces to

$$E_{n}(\theta )=f(\theta_{1})+f(\theta_{2})+K(1-\exp(-n{\left(\theta_{1}-\theta_{1}\right)}^{2}),$$

with *E*_0_(***θ***) = *f*(*θ*_1_) + *f*(*θ*_2_) and *K* a positive constant.

Consider now any sequence ***θ***^*n*^ such that ***θ***^*n*^ → ***θ*** and (***θ***^*n*^) ′ → ***θ*** ′ uniformly on [0, 1], such that, to fix ideas, ***θ***_0_ = (0, 0) and ***θ***_1_ = (1, 0): expanding the square of the first factor in the action functional *I*_*n*_ and letting $\rho_{n}(s)=\theta_{1}^{n}(s)-\theta_{2}^{n}(s)$ and $R_{n}(s)=2nK\rho_{n}(s)\exp(-n\rho_{n}^{2}(s))$, we find

$$|\nabla E_{n}(\theta^{n}(s)){|}^{2}={\left(f{\;}^{\prime }(\theta_{1}^{n}(s))+R_{n}(s)\right)}^{2}+{\left(f{\;}^{\prime }(\theta_{2}^{n}(s))-R_{n}(s)\right)}^{2}.$$

The idea is now to decompose the integration domain [0, 1] into the union of $I_{\delta }^{n}=\{s\in [0,1]:|\rho_{n}(s)|<\delta \}$ and its complement. On $[0,1]\setminus I_{\delta }^{n}$ it straightforward to see that *R*_*n*_ converges uniformly to zero, while on $I_{\delta }^{n}$ the sequence *R*_*n*_ is not bounded in the sup-norm and dominates the terms involving *f* ′. Hence, *I*_*n*_ can be approximated by

$$I_{n}(\theta^{n})∼\int_{I_{\delta }^{n}}\sqrt{2}|R_{n}(s)||(\theta^{n}){\;}^{\prime }(s)|\;\text{d}s+\int_{[0,1]\setminus I_{\delta }^{n}}|\nabla E_{0}(\theta^{n}(s))∥(\theta^{n}){\;}^{\prime }(s)|\;\text{d}s$$

for *n* large. Now, $\nabla E_{0}(\theta^{n}(s))$ and (***θ***^*n*^) ′(*s*) converge uniformly to $\nabla E_{0}(\theta (s))$ and ***θ*** ′(*s*) on [0, 1], and therefore, by the arbitrariness of *δ*, the second term above converges to ${\tilde{I}}_{0}(\theta )$. On the other hand, if we assume for simplicity that the only point at which *ρ*_*n*_(*s*) vanish is *s* = 0, on $I_{\delta }^{n}$ we have that $\rho_{n}(s)=\theta_{1}^{n}(s)-\theta_{2}^{n}(s)∼(c_{1}^{n}-c_{2}^{n})s$, with $c_{i}^{n}=(\theta_{i}^{n}){\;}^{\prime }(0)\to c_{i}=\theta_{i}{\;}^{\prime }(0)$, so that the first term above becomes, if *c*_1_ ≠ *c*_2_,

$$\begin{array}{rl} & \int_{I_{\delta }^{n}}2\sqrt{2}nK|c_{1}^{n}-c_{2}^{n}|s\;{\text{e}}^{-n{\left(c_{1}^{n}-c_{2}^{n}\right)}^{2}s^{2}}\sqrt{{\left(c_{1}^{n}\right)}^{2}+{\left(c_{2}^{n}\right)}^{2}}\;\text{d}s\\ & \;∼K\frac{\sqrt{2({\left(c_{1}^{n}\right)}^{2}+{\left(c_{2}^{n}\right)}^{2})}}{\left|c_{1}^{n}-c_{2}^{n}\right|}\to K\frac{\sqrt{2(c_{1}^{2}+c_{2}^{2})}}{\left|c_{1}-c_{2}\right|}, \end{array}$$

which is the function *Φ*(***θ***(0), ***θ*** ′(0)) in the expression for *I*_0_.

Third, we prove (c). Given that, as *b* = *n* → ∞ the MEP is approximately the straight line segment (A 2), we can compute the barriers by evaluating the energy on it:

$$\begin{array}{rl} E(t) & =f(t)+\sum_{\;j}(c_{1}A_{ij}+c_{2}B_{ij}+c_{3}C_{ij})(1-\exp(-a(t^{2}+\theta_{\;j}^{2})))\\ & \;+\sum_{\;j}(c_{4}C_{ij}+c_{5}\;{\tilde{A}}_{ij}H(t-\theta_{\;j})+c_{5}\;\;{\tilde{A}}_{\;ji}H(\theta_{\;j}-t))\\ & \;\times (1-\exp(-b{\left(t-\theta_{\;j}\right)}^{2}))+\;\text{const.}, \end{array}$$

where *H* is the Heaviside step function. Therefore,

A 4 $$\begin{array}{rl} E(t)-E(0) & =f(t)+\sum_{\;j/\theta_{\;j}=0}(c_{1}A_{ij}+c_{2}B_{ij}+c_{3}C_{ij})(1-\exp(-at^{2}))\\ & \;+\sum_{\;j/\theta_{\;j}=0}(c_{4}C_{ij}+c_{5}\;\;{\tilde{A}}_{ij})(1-\exp(-bt^{2}))\\ & \;-\sum_{\;j/\theta_{\;j}=1}(c_{4}C_{ij}+c_{5}\;\;{\tilde{A}}_{\;ji})(1-\exp(-b{\left(1-t\right)}^{2})). \end{array}$$

The above function is the sum of *f* and three monotone functions, two increasing and one decreasing. For *b* and *a* large enough, *E* has a single maximum in [0, 1], at the point at which *f* has its maximum, and the value of the maximum of *E* is exactly the sum of the maxima of its constituents, which yields the relation (3.4).

Finally, we list in table 2 the results of numerical simulations showing how the initial opening mode depends on the constants *c*_4_ and *c*_5_.
